# Supplementary figures and images for: Trypanosoma brucei rhodesiense Inhibitor of Cysteine Peptidase (ICP) Is Required for Virulence in Mice and to Attenuate the Inflammatory Response
Source: Int J Mol Sci. 2022 Dec 30;24(1):656. doi: 10.3390/ijms24010656 (PMC9820468; doi:10.3390/ijms24010656)

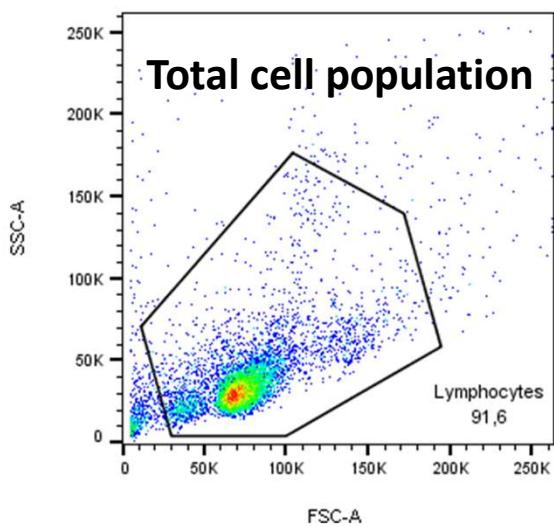

**CD4**

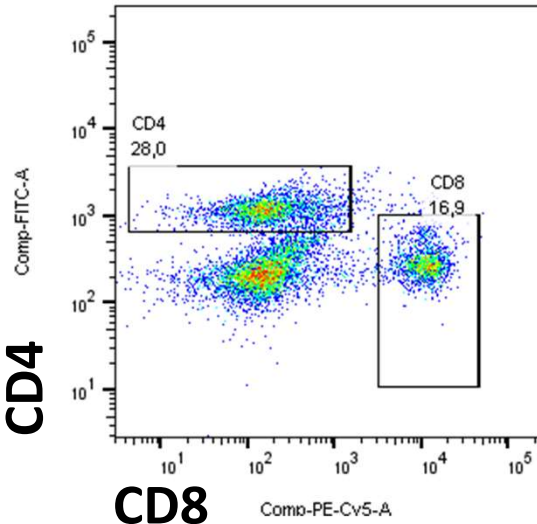

**CD8**

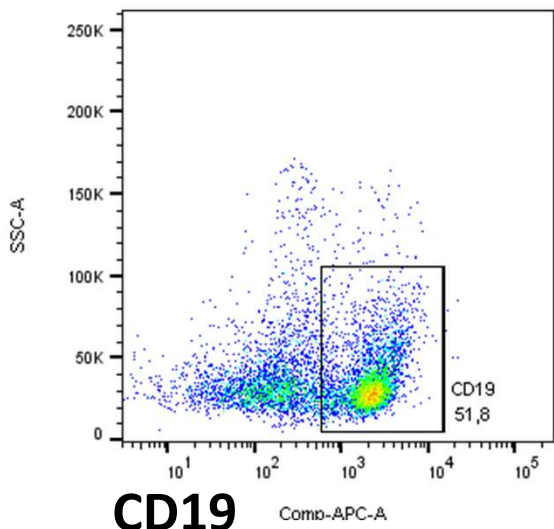

**CD19**

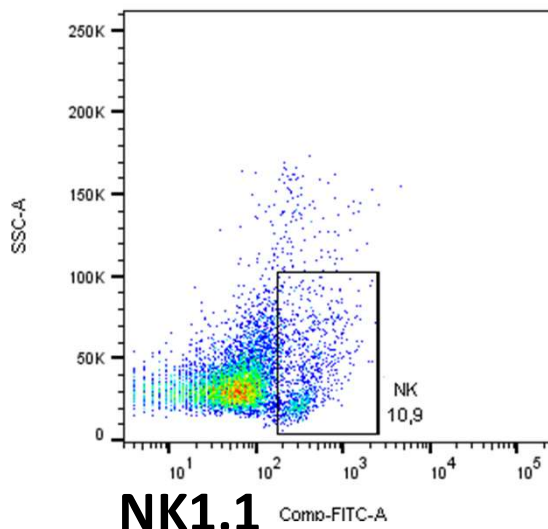

**NK1.1**

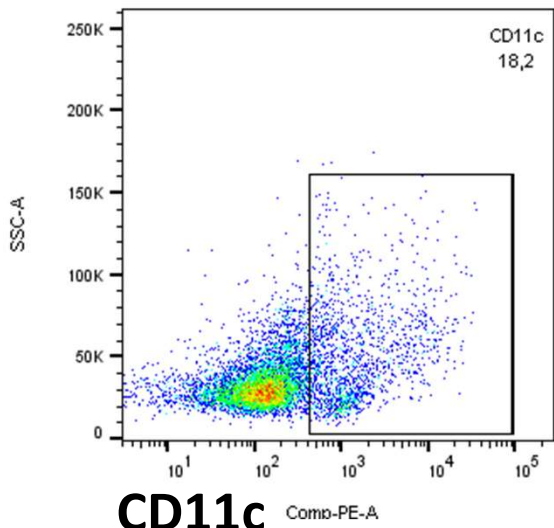

**CD11c**

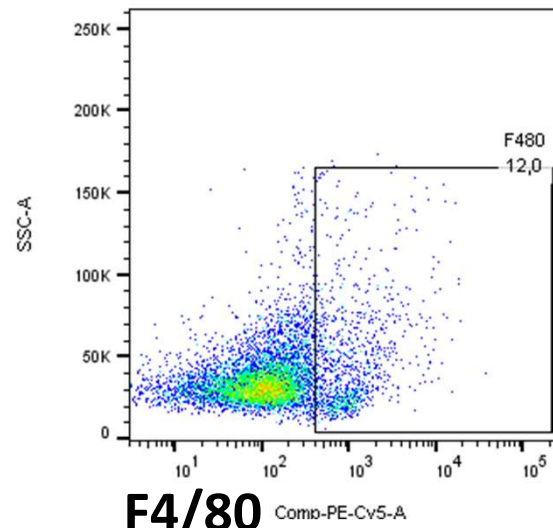

**F4/80**

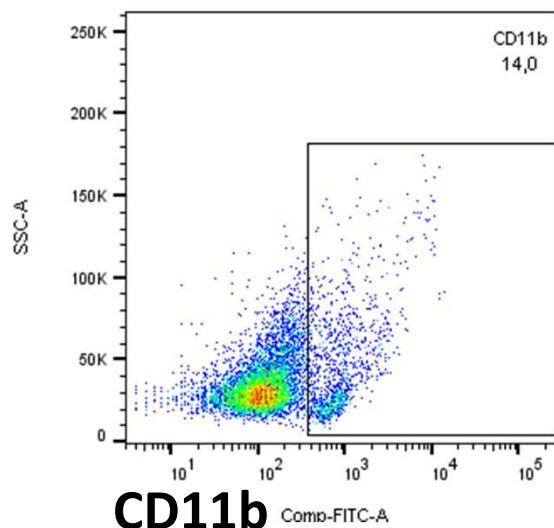

**CD11b**

**Ly6G**

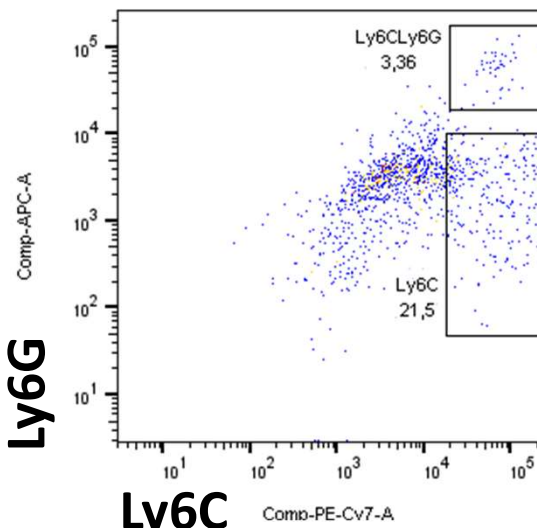

**Ly6C**

Supplement: Supplementary file 1 [file ijms-24-00656-s001.zip › ijms-2048618-Supplementary Figure S1.pdf]
